# *TP53* Mutations and *HBX* Status Analysis in Hepatocellular Carcinomas from Iran: Evidence for Lack of Association between HBV Genotype D and *TP53 R249S* Mutations

**DOI:** 10.1155/2011/475965

**Published:** 2011-08-17

**Authors:** Behnoush Abedi-Ardekani, Doriane Gouas, Stephanie Villar, Masoud Sotoudeh, Pierre Hainaut

**Affiliations:** ^1^Mechanisms of Carcinogenesis Section, Molecular Carcinogenesis Group, International Agency for Research on Cancer, 150 Cours Albert Thomas, 69372 Lyon, France; ^2^Digestive Disease Research Center, Shariati Hospital, Tehran University of Medical Sciences, Kargar Shomali Avenue, 14117 Tehran, Iran

## Abstract

High incidence of HCC is mostly due to the combination of two major risk factors, chronic infection with hepatitis B (HBV) and/or C (HCV) viruses and exposure to the mycotoxin aflatoxin B_1_, which induces a particular mutation at codon 249 in *TP53 (R249S)*. Eight genotypes of HBV are diversely found in high and low incidence areas. Regardless of documented strong associations between *TP53 R249S* mutation and HBV genotypes B, C, A or E, there is no report of such association for genotype D despite of the presence of aflatoxin in areas with high prevalence of HBV genotype D. In Iran, 3% of the population is chronically infected with HBV, predominantly genotype D. Twenty-one histologically confirmed HCC cases from Iran were analyzed for *TP53 R249S* and HBV double mutations 1762^T^/1764^A^, hallmarks of more pathogenic forms of HBV. We did not detect any of these mutations. In addition, we report the only case identified so far carrying both *R249S* mutation and chronic HBV genotype D, a patient from The Gambia in West Africa. This paper suggests that association between HBV genotype D and aflatoxin-induced *TP53* mutation is uncommon, explaining the relatively lower incidence of HCC in areas where genotype D is highly prevalent.

## 1. Introduction

Hepatocellular carcinoma (HCC) is the sixth most common cancer, accounting for about 5% of all human cancers and the second cause of cancer death in the world [1]. In 2008, an estimated of 748,000 new cases of liver cancer occurred and 696,000 people died of this cancer. Although liver cancer is a global health problem and a major cause of mortality and morbidity, low-income, tropical countries are more commonly affected, and 80% of cases occur in these regions, especially in South-East Asia and Sub-Sahara Africa [2]. HCC is the third most common cancer in China with the age-standardized rate (ASR) of 37.4 and 34.1 per 100,000 person-years in males and females, respectively. In Western Africa, the ASR of HCC is 16.6 in males and 16.5 per 100,000 person-years in females, where this cancer accounts for the second most common cancer [1]. Cancer risk is 2–7 times higher in men than in women but this ratio varies across the world. 

The most important risk factors for liver carcinogenesis include chronic infections with hepatitis B (HBV) and C (HCV) viruses, chronic alcohol consumption, and consumption of aflatoxin B_1_ (AFB_1_) contaminated food. With the presence of about 2 billion people with past or present HBV infection across the world and more than 350 million chronic carriers, HBV remains one of the most common human pathogens and a significant public health problem. Different mechanisms are suspected to be responsible for its role in liver carcinogenesis. HBV, a member of hepadnaviruses, has a partially double-stranded DNA genome containing four overlapping open reading frames: Pre-C/C, encoding the HBeAg and HBcAg; P, encoding the viral polymerase; Pre-S/S encoding the three viral surface proteins; and X, producing HBx protein. HBx is a 17.5 KDa, 154 amino acid multifunctional regulator protein that has been closely associated with HCC. It is known that HBx stimulates HBV replication and interferes with many cellular signaling pathways but its precise role in human liver carcinogenesis is still unclear. Furthermore, the integration of HBV in 80–90% of host genome of HBV-infected HCC cases has been reported, suggesting a mechanism of insertional mutagenesis. Several studies have shown that C-terminal truncation of integrated *HBX* may promote tumorigenesis [3–6].

HBV has a variable genome sequence and is currently classified in eight different genotypes (from A to H), the prevalence of which varies geographically. Genotypes B and C are the most prevalent HBV forms in high incidence areas in South-East Asia, while genotype E is common in West Africa and genotype D is the major genotype in eastern Africa, Middle East, Central Asia, and India [7]. The diverse oncogenicity of HBV genotypes has been studied by multiple researchers [6, 8, 9]. However, the impact of their role in molecular mechanisms of carcinogenesis has not been fully investigated yet. 

Epidemiological studies in high incidence areas indicate that dietary AFB_1_ contributes to the development of HCC and that the two main risk factors, HBV and AFB_1_, have a synergistic effect in liver carcinogenesis [10]. Aflatoxins are a group of mycotoxins that contaminate many sources of food in hot and humid countries such as West Africa, parts of China, South-East Asia, and regions of Latin America. The molecular hallmark of AFB_1_ intoxication in relation to HCC is a specific mutation at codon 249 of the *TP53* gene. This mutation is a single-base substitution at the third base of codon 249 (AGG to AGT), which replaces an arginine “R” by a serine “S”. This mutation has been reported in about 75% of HCC cases in high incidence areas (China or East Africa). Such a mutation is not detected in HCC cases from nonaflatoxin contaminated areas [11]. 

In addition, a double mutation in HBV genome corresponding to an adenine to thymine transversion at nucleotide 1762, and a guanine to adenine transition at nucleotide 1764 (1762^T^/1764^A^) has been reported as strongly associated with risk of HCC in high incidence areas of China, Thailand, or West Africa [12–15]. 

In this paper, we have analyzed HCC cases from Iran, a Middle East/Central Asian country of moderate incidence of HCC (ASR of 2.1 per 100,000 person-years in both sexes) despite the relatively high prevalence of chronic carriage of HBV, mainly genotype D [16–18], in the population and the document presence of aflatoxin, at least levels, in several components of the diet [19–21]. With the objective of documenting possible association between genotype D and molecular hallmarks of HCC detected in high incidence areas, we have analyzed *TP53 R249S* mutations and HBV double mutations. In addition, we are documenting one case of a patient from West Africa with HBV genotype D and *TP53 R249S* mutation. This study establishes that association between *TP53 R249S* and HBV genotype D is uncommon, providing a clue to explain the relatively modest incidence of HCC in regions where this genotype is the most common.

## 2. Materials and Methods

### 2.1. Case Selection

Thirty-four formalin fixed paraffin embedded (FFPE) HCC cases were selected from the pathology archives of two hospitals (Shariati and Atieh) and one day clinic (Tehran Gastroenterology and Hepatology Center) in Tehran, based on the diagnosis, size of the tissue, and the availability of the clinical data. All cases were histologically and immunohistochemically confirmed as primary HCC. Cases were rereviewed by a pathologist at the International Agency for Research on Cancer (BA-A) to confirm the diagnosis and to annotate tumor areas containing at least 70% of tumor cells. The study was conducted in accordance with international ethical standards applicable both in Iran and at IARC. In particular, cases were retrospectively selected from the pathology archives and were anonymized prior to analysis. The HCC case from The Gambia was identified in the context of a case-control study, the Gambia Liver Cancer Study (GLCS), the design and ethical approval of which has been previously described [22].

### 2.2. DNA Extraction

Sequential sections were prepared from selected FFPE blocks, and DNA was extracted by using QIAamp DNA MicroKit from QIAGEN (Hilden, Germany) as described by the manufacturer. The quantity of extracted DNA was measured by Nanodrop.

### 2.3. Detection of TP53 Mutations

For *TP53* mutations analysis, exon 7 (encompassing *R249S*) was amplified by PCR and directly sequenced. Sequencing was performed on an Applied Biosystems PRISM 3100 Genetic Analyzer (Applied Biosystems, Foster City, CA). Each PCR product was generated in duplicate, with one product sequenced in the forward direction and the other in the reverse direction. In the case of discordant sequencing results, the complementary sequences were analyzed and the final result was confirmed by sequencing a third, independent PCR product. Primers, PCR, and sequencing conditions are described in detail on the *TP53* IARC database website at http://www-p53.iarc.fr/. Chromatograms were analyzed semiautomatically by visual inspection of sequences imported in sequence analysis software Seqscape (Applied Biosystems) using the reference sequence NC_000017.9 from Genbank (http://www-p53.iarc.fr/TP53sequence_NC_000017-9.html). Sequencing results were reevaluated by restriction fragment length polymorphism (RFLP) with HaeIII, the cutting site of which (GGCC) is abrogated by *R249S* mutation (GTCC) [22].

### 2.4. Analysis of HBX Status and Mutations

The method used for* HBX* amplification is based on the method described by Ma and coworkers [5]. Four overlapping amplicons of progressive length, starting at the 5′ end of HBX sequence, were generated, the shorter one covering the first 139 bp and the longer one covering the whole gene sequence (425 bp). One common forward primer X1F was used in all PCR (5′-GGGACGTCCTTTGTCTACGT-3′), and four different reverse primers were used along *HBX* gene, X1R (5′-GGGAGACCGCGTAAAGAGAG-3′) (139 bp), X2R (5′-GTGCAGAGGTGAAGCGAAGT-3′) (192 bp), X3R (5′-CCCAACTCCTCCCAGTCTTT-3′) (334 bp), or X4R (5′-GGCAGAGGTGAAAAAGTTGCA-3′) (425 bp). Fifty cycles were performed for all reactions after activation of the GoTaq Hot Star Polymerase at 94°C for 2 min (0.5 U, Promega) (denaturing at 94°C for 45 sec, annealing at 62°C for 45 sec, and extension at 72°C for 45 sec) followed by extension at 72°C for 10 min. Mutation analysis of *HBX* gene was performed using nucleotide and protein alignment with GenBank references *HBX* genes of genotype D (the predominant genotype in Iran) with MEGA5 software [23].

### 2.5. HBS Amplification and Genotype Determination in a Patient from The Gambia

*HBS* gene was amplified from free-circulating plasma DNA using a heminested PCR. For the first reaction, 2 *μ*L of DNA were used with primers S_HBVpol1 (5′-CCTGCTGGTGGCTCCAGTTCA-3′) and S_HBVporv2 (5′-AAAGCCCAAAAGACCCACAAT-3′); PCR settings were 95°C (15 min); 40 cycles of 95°C (30 sec), 60°C (30 sec), 72°C (1 min); then 72°C for 7 min. Second step used 2 *μ*L of the first reaction and primers S_HBV123s (5′-TCGAGGATTGGGGACCCTG-3′) and S_HBVporv2 PCR settings were 95°C (15 min); 45 cycles of 95°C (30 s), 58°C (30 s), 72°C (1 min); then 72°C for 7 min. 5 *μ*L of PCR products were purified using standard ExoSap-IT (usb Corporation, Ohio, USA) enzyme, and nucleotide sequences were determined for both strands by automated dideoxy sequencing (sequencer AbiPrism 3100, Applied Biosystems, CA, USA). Direct sequencing on amplified fragments was performed using the primers: S_HBV123s, S_HBVporv2 and S_HBV778r (5′-GAGGTATAAAGGGACTCAAG-3′). HBV genotypes and subtypes were determined as previously described [24] and a phylogenetic tree was made using the software MEGA5 [23].

## 3. Results and Discussion

Of the initial 34 cases of archived HCC cases from Iran, exon 7 was successfully and reproducibly analyzed in 18 cases and *HBX* mutations were analyzed in 21 cases. Other samples could not be fully analyzed due to insufficient quality and/or low amount of extracted DNA.  Table 1 shows the characteristics of the 21 analyzed cases and the major findings. Mean age was 56.4(±19.3) years and all except one (5%) were men. HBV immunological data was available in the medical files of 15 (71.4%) cases, including 6 (28.6%) positive cases and 9 (42.8%) negative cases. Data was not available for the other 6. Cirrhosis was histologically confirmed in 7 subjects (33.3%), absent in 7 (33.3%), and no data was available for the rest (7; 33.3%). *HBX* was not present in one case (4.8%), complete in 7 (38.1%), and 3′-truncated in 12 (57.1%). Our results on *HBX* status showed a higher proportion of 3′-truncated *HBX*, concordant with a previous study which has reported a COOH-deletion in about 80% of HCC from China [5]. Moreover, of the 20 cases positive for *HBX* (complete or 3′-truncated) only 6 were positive by serology. Noteworthy, among the 6 cases without information about HBV serology, all but one were positive for *HBX*. Together, these observations suggest a relatively high prevalence of occult HBV infection.

 Both *TP53 R249S* mutations and double mutations 1762^T^/1764^A^ in HBV genome have been considered as hallmarks of aflatoxin-induced HCC in a context of HBV chronic infection. Kuang et al. reported the detection of these mutations in both plasma and tumor samples of HCC cases and most importantly, they were detected in some individuals up to 8 years before HCC diagnosis [13]. In this paper, we selected a small group of histologically confirmed HCC cases from Iran, where HCC is the 11th most common cancer and chronic HBV infection affects 3% of the population and is considered as a major health problem and probably the most common cause of HCC in this region. Mutation analysis of *TP53* and *HBX* did not show case positive for *R249S* or 1762^T^/1764^A^ mutation, suggesting that these two hallmarks of HCC were absent or infrequent in this particular etiological context.

**Figure 1:**
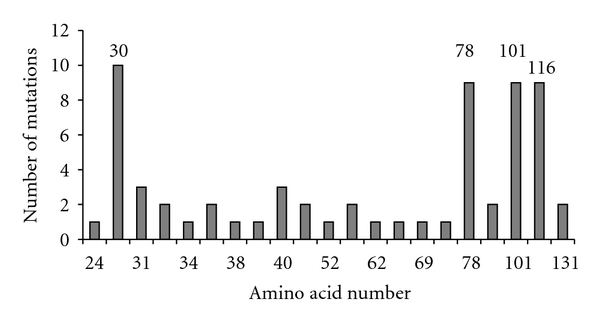
Number of mutations along of *HBx* amino acids. Mutation analysis was performed using alignment with GenBank HBV references by MEGA5 software [23]. Four main mutation points were amino acids 30, 78, 101, and 116.

The predominant HBV genotype in Iran is genotype D, which is different from the most common genotypes found in highest incidence areas (genotypes B and C in South-East Asia, genotypes E and A in West Africa, genotypes A, G, and F in Americas). The sample size of our study was small, and most of our samples were selected among needle biopsies with tiny pieces of tissues, which limited the success rate for amplifying DNA and obtain high quality DNA to perform a complete molecular analysis. For example, we failed to amplify *HBS* gene for genotype analysis of our cases, thus making it not possible to formally identify the genotype of these patients. We thus relied on previous publications from different parts of Iran, which detected genotype D as the predominant one [16–18]. Nevertheless, our paper is the first one that analyzed the molecular hallmarks of carcinogenesis in HCC cases in an area of high predominance of genotype D. The absence of double mutations 1762^T^/1764^A^ in HCC in Iran (Figure 1) shows that different HBV genotypes may undergo specific mutations in the process of hepatocarcinogenesis. The fact that genotype D does not appear to acquire nor accumulate the mutations that characterize the genotypes associated with HCC in areas of high incidence may explain at least in part the relatively low incidence of HCC in Iran and in other countries of high prevalence of genotype D despite relatively high chronic carriage of HBV in these populations. Interestingly, *HBX* mutation analysis identified mutations in amino acids that have not been noted in HBV genotypes from high incidence areas so far. These include mutations at amino acid numbers 30, 78, 101, and 116 (Figure 1). This finding may represent another molecular difference of liver carcinogenesis between high and low risk regions. Further studies are needed to understand the role of HBV genotypes and mutations that are less common in low incidence regions.

The absence of *TP53 R249S* mutations in HCC from Iran also suggests that the mechanisms of carcinogenesis involved are distinct from those that drive hepatocarcinogenesis in areas of highest incidence. Recent reports analyzing the presence of AFB_1_ in food and nuts from Iran have failed to show significant levels of fungal contamination [19–21]. Therefore, the population exposure to aflatoxin might be low, although aflatoxin contamination is detectable in many regions spanning the areas of high prevalence of the genotype D of HBV.

**Figure 2:**
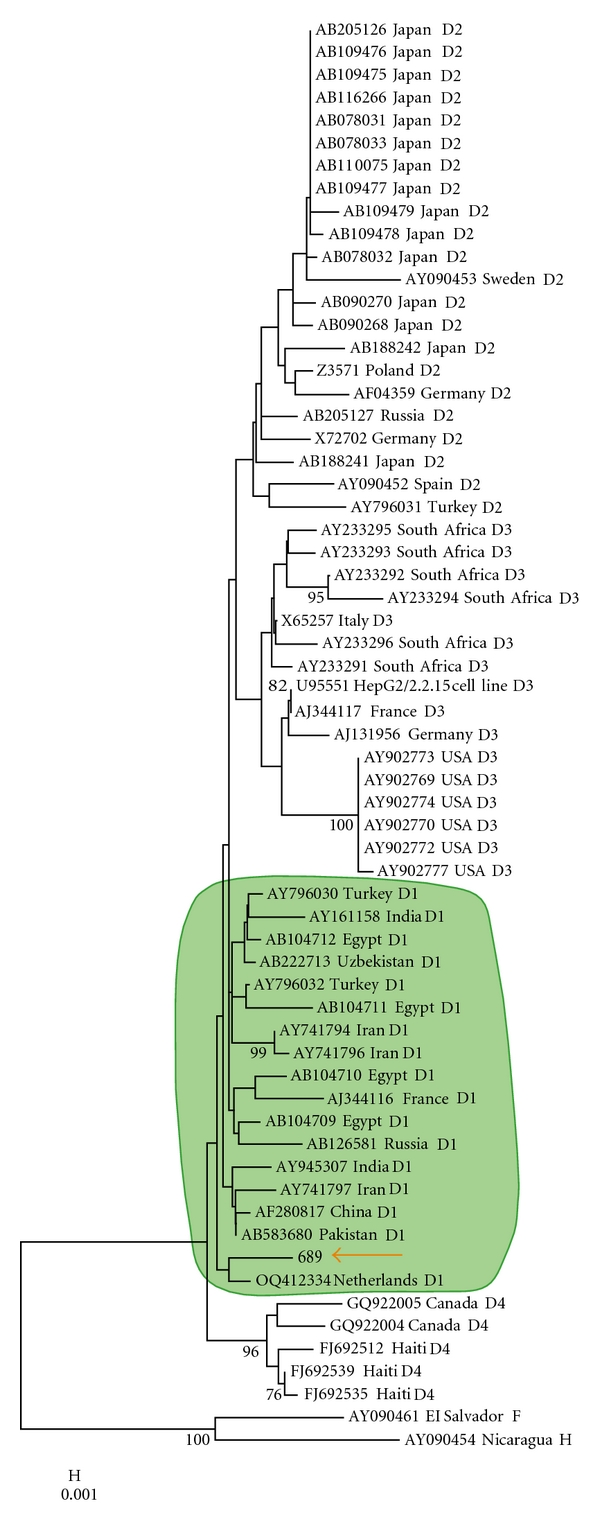
Neighbor-joining phylogenetic analysis using *HBS* gene of genotype D. The four subgenotypes D1 to D4 sequences were extracted from GenBank. The HCC *R249S* positive case from the case-control study from The Gambia is shown by an orange arrow and corresponds to subgenotype D1 (green circle). Made by the software MEGA5 [23].

The present paper highlights that genotype D and *R249S* mutations are not incompatible or mutually exclusive in HCC pathogenesis. Indeed, we report here a single HCC case from a previously described case-control study from The Gambia (West Africa) with HBV infection by genotype D1 (subtype *ayw2*) (Figure 2) and with the presence of the *R249S TP53* mutation [22, 25, 26]. This HCC case *R249S*-genotype D1 is the first one described so far having these molecular characteristics. The patient is a 45-year-old man without HBV double mutation. Several epidemiological studies have shown that the risk of HCC is increased in the presence of both HBV infection and AFB_1_ exposure. *R249S* mutation has been shown to be detectable in plasma DNA and tumor tissue of HCC cases in China and in The Gambia where AFB_1_ and HBV infection are prevalent with genotypes B, C, A, and E, respectively. However, this *R249S* mutation has never been reported in the presence of HBV genotype D even in India where predominance of HBV genotype D could be considered as the major risk factor for liver carcinogenesis possibly reinforced by the presence of AFB_1_ exposure [8, 11].

## 4. Conclusion

Our results on HCC cases from Iran, an area with HBV genotype D infection, failed to detect the molecular hallmarks of the etiologic role of HBV chronic infection and AFB_1_ exposure in liver carcinogenesis. Our findings are the starting point for further studies to evaluate and to discover the molecular mechanisms of HBV genotypes differently distributed in high-risk regions. In addition, we described here the first case of HCC in a patient from The Gambia with the *TP53 R249S* mutation and infection with HBV genotype D1, showing that, despite its relative rarity, the association between genotype D and *TP53 R249S* mutation may occasionally occur. Further studies are needed to understand the molecular mechanisms of synergy between HBV chronic infection and aflatoxin mutagenesis in order to explain why this association may operate more systematically with certain, but not all, HBV genotypes. Such difference might be of major importance to evaluate the impact of risk factors on the incidence of HCC in different parts of the world.

##  Conflict of Interests

The authors declare that there is no conflict of interests.

## Figures and Tables

**Table 1:** Characteristics and molecular status of 21 HCC cases from Iran.

Characteristics	No. (%)
Mean age (±SD)	56.4 (±19.3)
Sex	
Women	1 (4.8)
Men	20 (95.2)
HBV status	
Positive	6 (28.6)
Negative	9 (42.8)
N/A	6 (28.6)
Cirrhosis	
Present	7 (33.3)
Absent	7 (33.3)
N/A	7 (33.3)
*TP53* mutation (*R249S*)	
Absent	21 (100.0)
Present	0 (0.0)
HBV Double mutation (1762^T^/1764^A^)	
Absent	21 (100.0)
Present	0 (0.0)
*HBX* status	
Complete	8 (38.1)
3′-Truncated	12 (57.1)
Absent	1 (4.8)

N/A: Not available.
